# Increased tooth mobility after fixed orthodontic appliance treatment can be selectively utilized for case refinement via positioner therapy - a pilot study

**DOI:** 10.1186/s12903-020-01097-4

**Published:** 2020-04-16

**Authors:** L. Keilig, J. Goedecke, C. Bourauel, N. Daratsianos, C. Dirk, A. Jäger, A. Konermann

**Affiliations:** 1 Endowed Professorship for Oral Medical Technology, University of Bonn, Bonn, Germany; 2 Department of Orthodontics, University of Bonn, Welschnonnenstr. 17, 53111 Bonn, Germany

**Keywords:** Case refinement, Orthodontic tooth movement, Positioner, Tooth mobility measurement

## Abstract

**Background:**

Increased tooth mobility persists after fixed orthodontic appliance removal, which is therapeutically utilized for post-treatment finishing with positioners. As such a fine adjustment is only required for selected teeth, the aim of this pilot study was to investigate tooth mobility in vivo on corrected and uncorrected subgroups under positioner therapy.

**Methods:**

Mobility was measured on upper teeth of 10 patients (mean age 16.8) by applying loadings for 0.1, 1.0 and 10.0 s with a novel device directly after multibracket appliance debonding as much as 2d, 1, 2 and 6 weeks later. Positioners were inserted at day 2. Specimens were divided into Group C (teeth corrected via positioner), Group N (uncorrected teeth adjacent to teeth from group C), and Group U (uncorrected teeth in an anchorage block). Untreated individuals served as controls (*n* = 10, mean age 22.4). Statistics were performed via Kolmogorov-Smirnov test and Welch’s unequal variances *t*-test for comparisons between groups. *P* < 0.05 was considered statistically significant.

**Results:**

After 1 week, tooth mobility in Group U almost resembled controls (13.0–15.7 N), and reached physiological values after 6 weeks (17.4 N vs. 17.3 N in controls). Group C (9.0–13.4 N) and Group N (9.2–14.7 N) maintained increased mobility after 6 weeks. Tooth mobility was generally higher by reason of long loading durations (10.0 s).

**Conclusions:**

Positioner therapy can selectively utilized increased tooth mobility upon orthodontic fixed appliance treatment for case refinements. Here, uncorrected teeth in anchorage blocks are not entailed by unwanted side effects and recover after 6 weeks post treatment. Corrected teeth and their neighbors exhibit enhanced mobility even after 6 weeks, which represents a necessity for the proper correction of tooth position, and concurrently arouses the requirement for an adequate retention protocol.

## Background

Orthodontic tooth movement via multibracket appliance underlies permanent tensile and compression strains within the periodontal ligament (PDL) and the surrounding bone [1]. The resulting remodeling processes lead to a widening of the alveolae, a reduced stiffness of the PDL and finally a shift of teeth accompanied by a required increased mobility and thus decreased restoring force [2]. These orthodontically induced effects and the heightened tooth mobility still persist a certain period after appliance removal, whereat post-treatment mobility reduction exhibits inter-individual differences [3]. The positioner is a removable, resilient orthodontic device for post-treatment finishing of orthodontic cases utilizing this phenomenon as treatment concept [4]. The positioner is constructed over a predetermined, ideal tooth ‘set-up’ and thus guides selected teeth into a more desired configuration by producing small amounts of detailed tooth movements through the material elasticity while minimizing undesirable side effects [5]. Most fine adjustments in tooth position occur within the first days after appliance insertion, however precise data on the time period for achievement of treatment aims are still missing. As only some teeth need fine corrections after fixed appliance treatment, it can be assumed that corrected and uncorrected teeth differ in the timing for restoration of the PDL and surrounding tissues as much as for the retrieval of physiological post-treatment tooth mobility (TM). This issue is a matter of debate that needs further investigation, even though the biomechanical properties of PDL tissues and TM have been approached in numerous in vitro and in vivo studies [1, 6–13]. The key restriction of all these attempts was the inability to measure tooth deflections with varying load velocities in order to accommodate the time dependence of the PDL due to hydrodynamics. These viscoelastic properties of the PDL arise during tooth displacement as periodontal liquids relocate and transpose with the surrounding [14]. Here, fast loadings lead to minor tooth displacements compared to slow loadings, as fluid exchange requires a longer time span [14].

Recently, a novel intraoral loading device (ILD) was introduced, which records tooth displacements in vivo with high resolution and monitors the time-dependent biomechanical behavior of the PDL [15]. Crown deflections of 0.2 mm according to the PDL thickness are applied and resulting forces are continuously measured with a resolution of 0.05 N.

The aim of this in vivo investigation was to monitor TM with the ILD and to identify time-dependent changes under positioner therapy after fixed orthodontic appliance treatment. More precisely, this pilot project should address whether there is a difference in the retrieval of physiological TM among a) teeth that are corrected via positioner, b) their adjacent, uncorrected teeth and c) uncorrected teeth in an anchorage block compared to untreated controls. Hence, the assumption that these subgroups differ in the time span for reestablishment of physiological TM should be elucidated, based on the hypothesis that applied forces exclusively affect the teeth to be corrected.

## Methods

### Ethics approval and consent to participate

The study was performed in full accordance with ethical principles, including the World Medical Association Declaration of Helsinki. The Ethical Committee of the University of Bonn gave Institutional Review Board approval for the study (181/13 as an extension to 030/12). Experimentation was undertaken with the understanding and written consent of each subject and parental consent for patients with age under 16, respectively.

### Aim, design and setting of the study

**Fig. 1:**
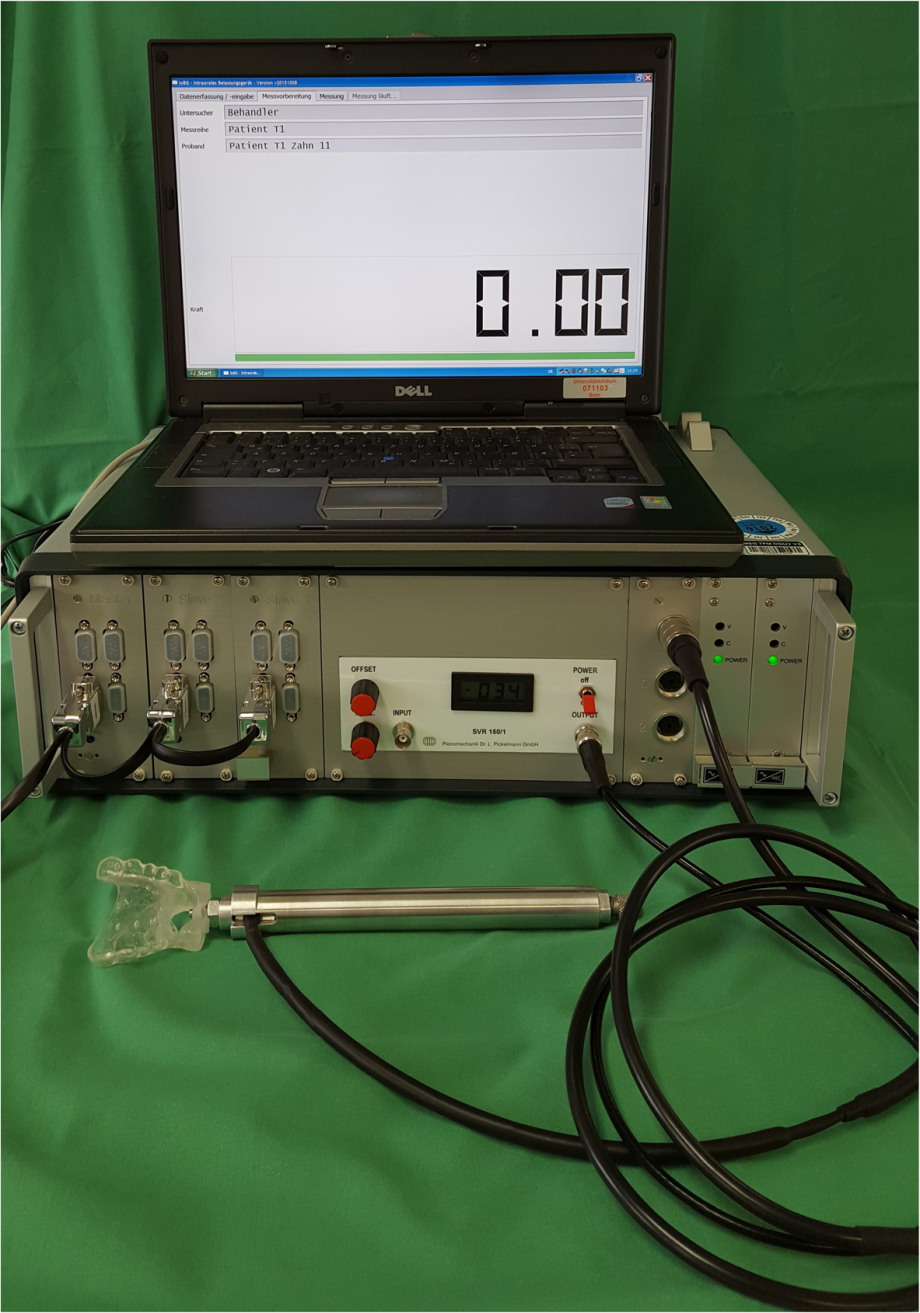
Intraoral loading device (ILD). ILD for in vivo measurements of tooth mobility with splint for fixation of the device on the patient’s upper jaw

The aim of this in vivo investigation was to monitor TM and to identify time-dependent changes under positioner therapy after fixed orthodontic appliance treatment. Measurement of TM was operated with the ILD, which is presented in Fig. 1. For intraoral device fixation individual splints for the upper jaw were constructed for each patient as specified elsewhere [15]. Measurements were conducted by displacing the centre of the anatomic crown in labio-lingual direction and recording the resulting forces simultaneously. During the loading phase, the tooth was displaced linearly from zero up to 0.2 mm over a time period of 0.1 s, 1.0 s, or 10.0 s. During the unloading phase, tooth displacement was reduced linearly back to zero over the same time period. After each single measurement, a pause of at least 1 min was maintained to guarantee relaxation of the PDL, normalization of the hydrodynamics of the fluid phase and realignment of the tooth to its initial position. Here, high TM values are reflected by low force values and vice versa.

Measurements in the treatment group were performed at 5 time points: T1 - directly after debonding of the fixed multibracket appliance, T2–2 d after debonding, T3–7 d after debonding, T4–14 d after debonding, T5–6 weeks after debonding. At T2, the positioner was inserted and patients were instructed to wear the appliance for a time period of 6 weeks for 14 h/d. The positioners were set up according to standard procedures and the corrected teeth were chosen pursuant to the individual refinement needs of each patient. For each positioner patient, 3 teeth were individually chosen for the measurements and assigned to one of three groups according to the following criteria:

- Group C: corrected tooth, whose position is actively modified via positioner
- Group N: uncorrected tooth adjacent to C, whose position is not modified via positioner
- Group U: uncorrected tooth not adjacent to C, whose position is not modified via positioner

Measurements in the control group were performed at a single time point on the right upper central incisor as reference.

### Treatment and control group

The treatment group incorporated 10 female patients (mean age 16.8 years) finishing orthodontic therapy with fixed multibracket appliances (022 slot size), who had the indication for case refinement in the upper jaw with a positioner. The control group incorporated 10 individuals (mean age 22.4 years) without orthodontic treatment, serving as reference of physiological TM for the values recorded in this study. Patient selection criteria comprised good general health, good oral hygiene, no medication affecting bone or soft tissue metabolism, no prosthetic restorations on the measured tooth, no premature occlusal contact on the front teeth, no radiographic signs of horizontal bone loss or vertical bony defects and no manifestations of root resorptions.

### Statistics

Previous to statistical analysis, the maximum force (Fmax; N) was extracted from the measured force/deflection curves for each single measurement. Quantitative descriptive statistics were performed with Microsoft Excel 2010 on these data to calculate means of Fmax and standard deviations for each time point (T1-T5), each loading duration (0.1 s, 1.0 s, 10.0 s), and each measured tooth group (C, N, U). Statistics were performed with Microsoft Excel 2010 and IBM SPSS 22. Normality distribution was assessed via Kolmogorov-Smirnov test. For comparisons between values from different groups, Welch’s unequal variances *t*-test was applied. Data are expressed as average ± SD. *P* < 0.05 was considered statistically significant.

## Results

Directly after removal of the multibracket appliance (T1), TM was markedly increased in all groups (C, N, U) compared to untreated controls. Mean Fmax control values ranged between 13.2 N and 17.3 N, whereas values of Group C and Group N resembled each other with magnitudes around 10.0 N. Data of Group U approximated control values at most with 13.0 N in the mean.

At T2, Group N and Group U already exhibited reduced TM ranging between 11.4 to 14.3 N and 11.8 to 14.7 N, respectively. Contrarily, Group C did not markedly change compared to T1 with 8.9 bis 11.4 N. At T3, Group U almost resembled control values with 13.0 to 15.7 N. Strikingly, TM in Group C and Group N decreased again below reference values. Here, TM in Group C was as high as directly after multibracket appliance removal. This phenomenon of force decline appeared at T4 for Group U as well with values of 11.0 to 14.7 N, being almost identical with the measurements of T2. At antipodes, Group C and N exhibited decreased TM with maximum forces of 12.0 N for Group C and 13.0 N for Group N at this time point. At T5, values of Group U reached up to 17.4 N and resembled physiological tooth mobility values of the control group with 17.3 N. Group C with 9.0 to 13.4 N and Group N with 9.2 to 14.7 N were still below physiological values.

Maximum forces resulting after long loading durations (10.0 s) were generally lower than short loading durations (0.1 s; 1.0 s), regardless of the time point of investigation. Table 1 illustrates the maximum force values for each group, loading duration and measurement time point.

**Table 1:** Mean maximum forces (N)

Loading duration	Measurement time point	Mean maximum force F_max (N) ± SD			
		Group C	Group N	Group U	Control group
**0.1 s**	T1	8.4 (± 2.7)	9.6 (± 3.4)	12.8 (± 5.7)	17.3 (± 6.0)
	T2	11.4 (± 3.7)	14.3 (± 5.5)	14.3 (± 5.9)	17.3 (± 6.0)
	T3	10.5 (± 5.1)	11.6 (± 6.2)	15.4 (± 7.5)	17.3 (± 6.0)
	T4	12.3 (± 4.0)	12.3 (± 4.9)	14.7 (± 8.0)	17.3 (± 6.0)
	T5	13.4 (± 4.1)	14.7 (± 4.3)	17.4 (± 6.0)	17.3 (± 6.0)
**1.0 s**	T1	10.1 (± 4.5)	10.8 (± 5.2)	13.0 (± 6.8)	15.8 (± 7.2)
	T2	11.3 (± 4.7)	13.6 (± 5.6)	14.7 (± 7.5)	15.8 (± 7.2)
	T3	9.7 (± 6.4)	12.3 (± 7.5)	15.7 (± 7.7)	15.8 (± 7.2)
	T4	11.3 (± 4.4)	13.0 (± 5.9)	14.2 (± 7.0)	15.8 (± 7.2)
	T5	12.0 (± 3.3)	12.9 (± 6.1)	15.4 (± 6.7)	15.8 (± 7.2)
**10.0 s**	T1	7.0 (± 2.2)	8.3 (± 4.1)	10.7 (± 6.2)	13.2 (± 6.2)
	T2	8.9 (± 3.7)	11.4(± 5.1)	11.8 (± 5.5)	13.2 (± 6.2)
	T3	8.3 (± 4.1)	8.7 (± 6.5)	13.0 (± 6.1)	13.2 (± 6.2)
	T4	8.9 (± 3.3)	9.3 (± 4.1)	11.0 (± 5.3)	13.2 (± 6.2)
	T5	9.0 (± 2.3)	9.2 (± 3.8)	12.0 (± 4.6)	13.2 (± 6.2)

Mean maximum forces (N) ± SD for the three different loading durations 0.1 s, 1.0 s and 10.0 s in the course of the measurement time points T1-T5. Group C represents corrected teeth, whose position was actively modified with the positioner, Group N represents uncorrected teeth adjacent to the ones of Group C, and Group U incorporates uncorrected teeth not adjacent to the ones from Group C

**Fig. 2:**
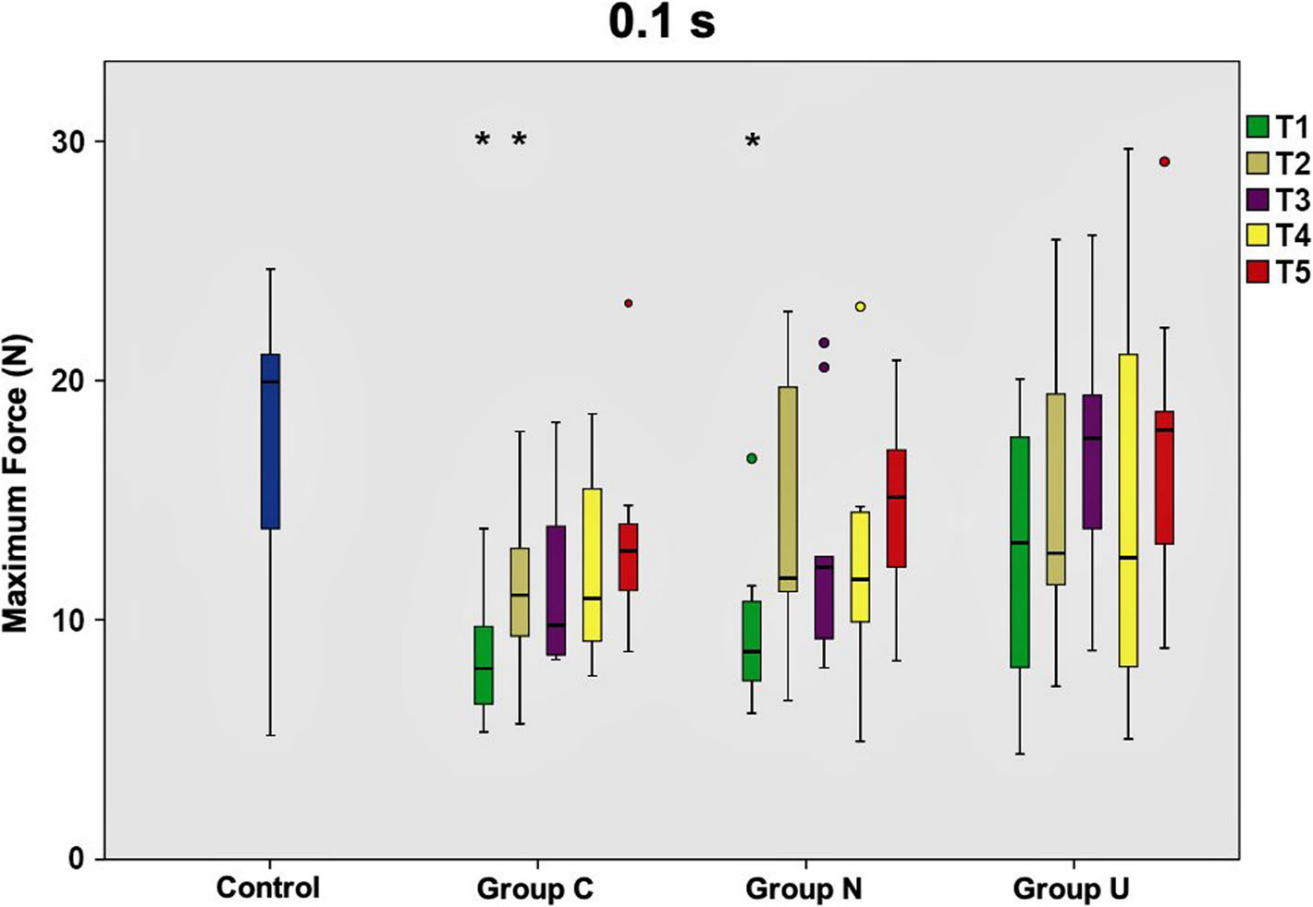
Maximum force values of tooth mobility measurements at 0.1 s loading duration. Mean maximum force values and standard deviations calculated from the tooth mobility measurements of the total patient collective (*n* = 10) at 0.1 s loading duration for all time points (T1-T5) investigated. Box plots represent the investigated subgroups and are compared to untreated controls. Group C - corrected teeth, whose position was actively modified with the positioner; Group N - uncorrected teeth adjacent to the ones of Group C; Group U - uncorrected teeth not adjacent to the ones from Group C. Statistically significant differences to controls were seen for Group C at T1 and T2, as much as for the teeth in Group N at T1 (*P* < 0.05)

**Fig. 3:**
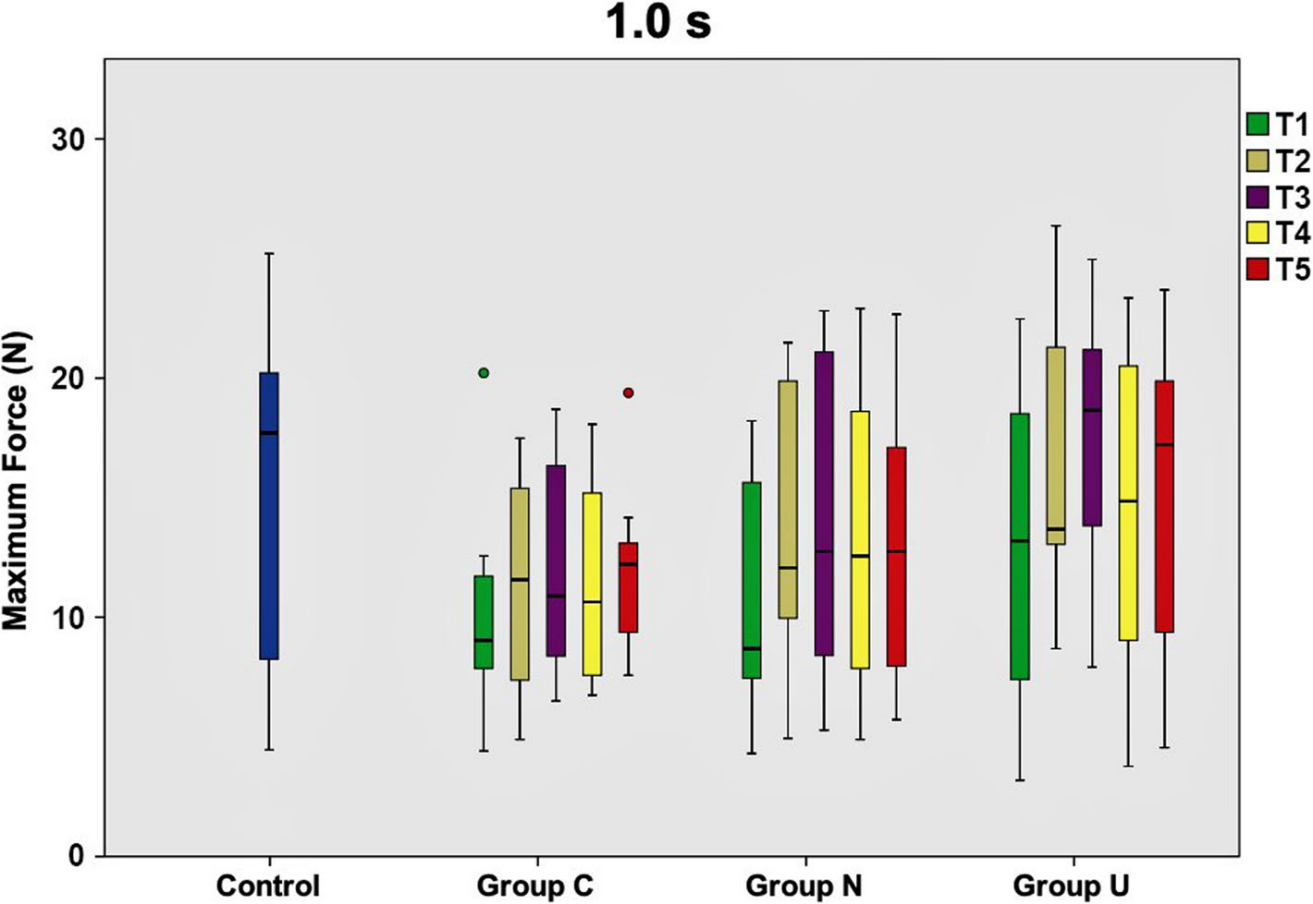
Maximum force values of tooth mobility measurements at 1.0 s loading duration. Mean maximum force values and standard deviations calculated from the tooth mobility measurements of the total patient collective (*n* = 10) at 1.0 s loading duration for all time points (T1-T5) investigated. Box plots represent the investigated subgroups and are compared to untreated controls. Group C - corrected teeth, whose position was actively modified with the positioner; Group N - uncorrected teeth adjacent to the ones of Group C; Group U - uncorrected teeth not adjacent to the ones from Group C

**Fig. 4:**
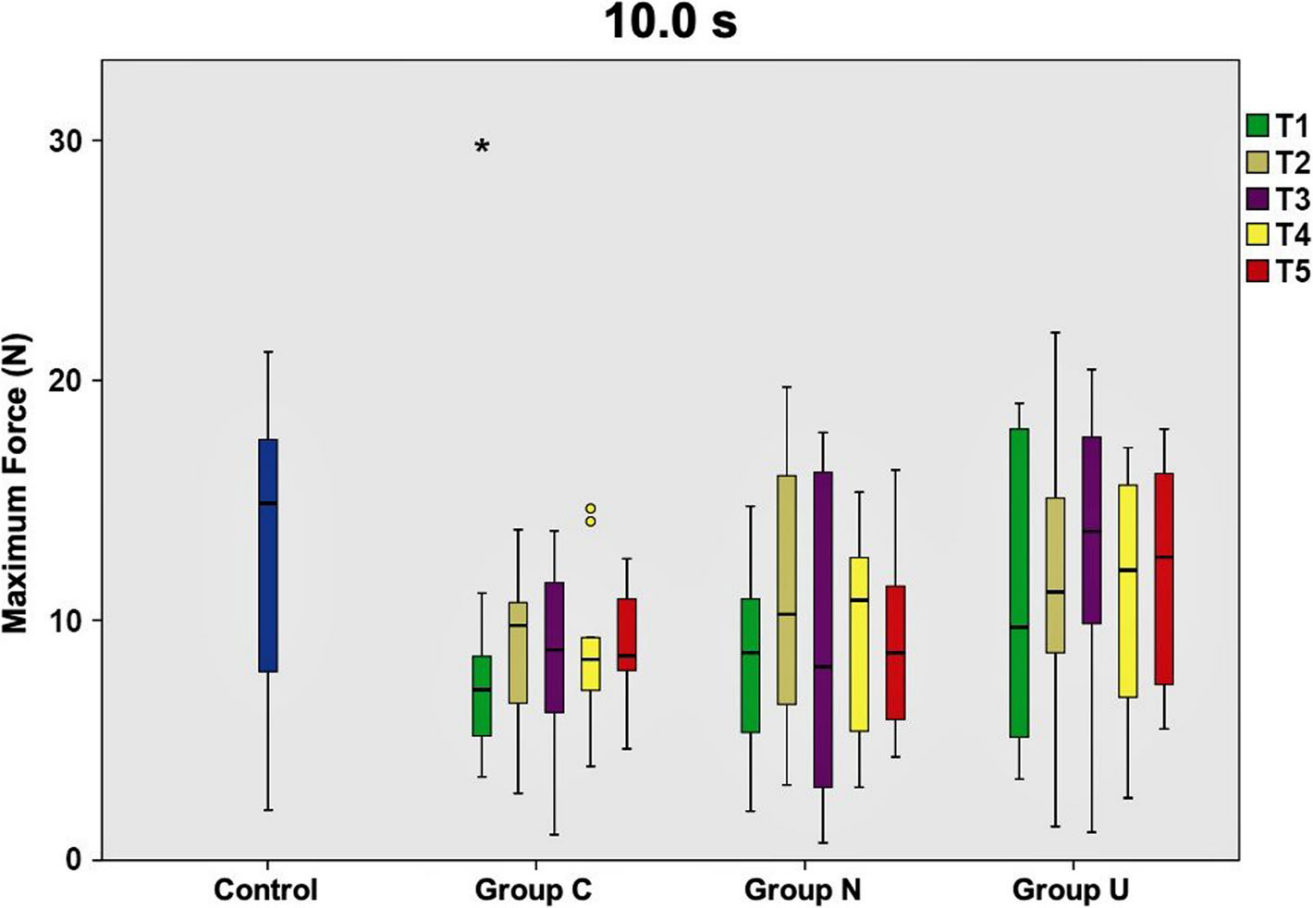
Maximum force values of tooth mobility measurements at 10.0 s loading duration. Mean maximum force values and standard deviations calculated from the tooth mobility measurements of the total patient collective (*n* = 10) at 10.0 s loading duration for all time points (T1-T5) investigated. Box plots represent the investigated subgroups and are compared to untreated controls. Group C - corrected teeth, whose position was actively modified with the positioner; Group N - uncorrected teeth adjacent to the ones of Group C; Group U - uncorrected teeth not adjacent to the ones from Group C. Statistically significant differences to controls were seen for Group C at T1 (*P* < 0.05)

When comparing the three experimental groups with the control group at a loading duration of 0.1 s, only Group U rapidly conformed to physiological force values, which was clearly evident at T5. On the contrary, force values of Group C and Group N were still far away from physiological values at T5. Statistically significant differences to controls were seen for Group C at T1 and T2, as much as for the teeth in Group N at T1. Data are graphically illustrated in Fig. 2. At a loading duration of 1.0 s, the pattern seen for 0.1 s could be reassured. Exclusively Group U reached physiological force values, but even earlier at T3. Fig. 3 collectively shows the measurements for 1.0 s. Loading durations of 10.0 s exhibited the same scheme for Group C and N as seen for the short loading durations. Also recurring is the fact that the teeth in Group U feature values equaling the controls at T3, however for this loading duration maximum force values decrease again up to T5, with 1.0 N below the value recorded at T3. Statistically significant differences to controls were seen for Group C at T1, which is presented in Fig. 4.

## Discussion

In this pilot study, TM was analyzed on upper teeth during positioner therapy for case refinement of selected multibracket cases, and compared to physiological values of untreated control teeth. Potential differences were expected in the retrieval of physiological TM among three different entities, namely a) teeth corrected via positioner, b) their adjacent, uncorrected teeth and c) uncorrected teeth in an anchorage block. Based on the hypothesis that applied forces exclusively affect the teeth to be corrected, it was assumed that uncorrected subgroups need a shorter time span for recovery of the PDL and reestablishment of physiological TM. TM was investigated in vivo with the ILD, which can effect tooth displacements with crown deflections of 0.2 mm according to the PDL thickness, and monitor the time-dependent biomechanical behavior of the PDL [15]. Previous works also tried to elucidate these aspects, but were limited by exclusive in vitro application, by inadequate handling for in vivo use, or most strikingly by the incapacity of monitoring tooth deflections with varying load velocities and thus the key characteristic of the PDL, the time dependence due to hydrodynamics [1, 6–13]. The ILD used in this study non-invasively records full force/deflection characteristics of teeth over a wide range of displacement velocities, from quasi-static loading to short-term pulses down to 0.1 s.

In our investigations, significant inter-individual differences could be observed for all maximum force data sets that have to be attributed to tooth anatomy. Investigations have shown that tooth mobility is influenced by the overall root surface area tangent to bone, at which roots with a larger volume effectuate reduced TM [16]. As the teeth to be corrected with the positioner were chosen pursuant to the individual refinement needs of each patient, Groups C, N and U incorporated incisors, canines and praemolars, exhibiting different root morphologies. This phenomenon has to be investigated in a future study on a larger patient collective with a sample size based on a power calculation with these data. Subsequent analyses on a larger cohort will also adjust the fact that the present data exhibit limitations due to the small sample size of this pilot project, potentially discovering subtle, but yet undetected distinctions.

Our analyses revealed a markedly increased TM, reflected by low force values directly after multibracket appliance removal for all teeth investigated, which is in accordance with previous results [2]. Thereupon, TM patterns began to vary over time, depending on the therapeutic subgroups of the positioner. The group of uncorrected teeth located in an anchorage block and not adjacent to a corrected one (Group U) retrieved almost physiological TM values after 7 days multibracket debonding, and reached control values after 6 weeks. Consequently, positioner therapy appears to be a controllable treatment appliance without impeding PDL recovery by exerting unwanted forces on teeth in an anchorage block. This rapid regeneration of the PDL within some weeks cannot be explained by bone remodeling processes that involve several months [17, 18], but rather seems to be induced by an alteration in the biomechanical behavior of the PDL. This aspect needs further investigation in subsequent in vitro experimentations to understand the underlying molecular mechanisms.

Contrarily to the teeth located in anchorage blocks, teeth without planned correction adjacent to corrected teeth (Group N) exhibited heightened TM and reduced force values even after 6 weeks post debonding. Thus, movements induced via positioner additionally seem to affect their adjacent teeth, with force exertion either by the appliance itself or by the approximal contacts of the teeth to be corrected. Both corrected teeth and their neighbors featured similar TM values over the whole time investigated. This knowledge about prolonged tooth mobility under positioner therapy can be taken advantage of for post-treatment finishing of orthodontic cases when longer time spans are needed for finalizing difficult cases. Moreover, this fact raises the necessity of valid retention after positioner therapy in order to maintain the treatment outcome.

Our results additionally prove the time-dependent, non-linear biomechanical behavior of the PDL, showing that TM increases at longer loading durations [14, 19]. As described earlier, these viscoelastic properties are aroused by the relocation of periodontal liquids, which requires a longer time span during fast loadings, thus leading to minor tooth displacements compared to slow loadings, and vice versa [14].

## Conclusions

Positioner therapy for case refinement of selected teeth after fixed orthodontic appliance therapy selectively exerts forces and does not entail unwanted side effects on uncorrected teeth in anchorage blocks. Based on the data of this work, further in vivo investigations as much as numerical analyses can be performed in order to make the impact and the consequences of orthodontic forces via positioner more predictable and thus reduce treatment side effects.

## Data Availability

The datasets used and analyzed during the current study are available from the corresponding author on reasonable request.

## Abbreviations

Fmax: maximum force
ILD: Intraoral loading device
PDL: Periodontal ligament
TM: Tooth mobility
